# Prognosis of Acute Kidney Injury and Hepatorenal Syndrome in Patients with Cirrhosis: A Prospective Cohort Study

**DOI:** 10.1155/2015/108139

**Published:** 2015-07-22

**Authors:** Andrew S. Allegretti, Guillermo Ortiz, Julia Wenger, Joseph J. Deferio, Joshua Wibecan, Sahir Kalim, Hector Tamez, Raymond T. Chung, S. Ananth Karumanchi, Ravi I. Thadhani

**Affiliations:** ^1^Division of Nephrology, Department of Medicine, Massachusetts General Hospital, Boston, MA 02114, USA; ^2^Division of Cardiology, Department of Medicine, Beth Israel Deaconess Medical Center, Boston, MA 02114, USA; ^3^Liver Center and Gastrointestinal Division, Department of Medicine, Massachusetts General Hospital, Boston, MA 02114, USA; ^4^Division of Nephrology, Department of Medicine, Beth Israel Deaconess Medical Center, Boston, MA 02215, USA

## Abstract

*Background/Aims*. Acute kidney injury is a common problem for patients with cirrhosis and is associated with poor survival. We aimed to examine the association between type of acute kidney injury and 90-day mortality. *Methods*. Prospective cohort study at a major US liver transplant center. A nephrologist's review of the urinary sediment was used in conjunction with the 2007 Ascites Club Criteria to stratify acute kidney injury into four groups: prerenal azotemia, hepatorenal syndrome, acute tubular necrosis, or other. *Results*. 120 participants with cirrhosis and acute kidney injury were analyzed. Ninety-day mortality was 14/40 (35%) with prerenal azotemia, 20/35 (57%) with hepatorenal syndrome, 21/36 (58%) with acute tubular necrosis, and 1/9 (11%) with other (*p* = 0.04 overall). Mortality was the same in hepatorenal syndrome compared to acute tubular necrosis (*p* = 0.99). Mortality was lower in prerenal azotemia compared to hepatorenal syndrome (*p* = 0.05) and acute tubular necrosis (*p* = 0.04). Ten participants (22%) were reclassified from hepatorenal syndrome to acute tubular necrosis because of granular casts on urinary sediment. *Conclusions*. Hepatorenal syndrome and acute tubular necrosis result in similar 90-day mortality. Review of urinary sediment may add important diagnostic information to this population. Multicenter studies are needed to validate these findings and better guide management.

## 1. Introduction

Acute kidney injury (AKI) is a common and life-threatening problem for patients with cirrhosis [1–3]. The differential diagnosis for AKI in this population is large. The most common etiologies are prerenal azotemia (PRA), acute tubular necrosis (ATN), and hepatorenal syndrome (HRS), but other causes such as glomerulonephritis, medication toxicity, and abdominal compartment syndrome from tense ascites occur as well [3, 4]. Regardless of etiology, AKI is associated with reduced survival [5–7]. Measures of renal function (i.e., serum creatinine) are factored prominently into prognostic scores such as the Model for End-Stage Liver Disease (MELD) and Chronic Liver Failure-Sequential Organ Failure Assessment (CLIF-SOFA) and have major implications for liver transplant allocation [8–10].

One cause of AKI unique to liver disease is HRS. Hepatorenal syndrome is thought to be due to splanchnic vasodilation causing hormonal imbalances that ultimately result in renal vasoconstriction and impaired renal function [4, 11]. Several studies suggest that HRS is associated with the highest mortality of all types of AKI [12–14]. As such, a diagnosis of HRS-related AKI is felt to have greater clinical significance over other types of AKI. Despite this, there is no definitive test for HRS, which remains a challenging diagnosis for clinicians.

Over the last several years, consensus guidelines have evolved to aid in the diagnosis of AKI in cirrhosis. The diagnostic criteria for HRS have been updated several times, most recently in 2007 [14, 15]. The definition of AKI itself has been debated as well, as some of the older definitions of AKI that used a doubling of serum creatinine or a preset threshold value had limited sensitivity to detect AKI in patients with cirrhosis [16, 17]. Recent studies support using the Acute Kidney Injury Network (AKIN) definition of AKI, which correlates closely with mortality in cirrhosis [17–23]. We sought to evaluate whether the type of AKI influenced the 90-day mortality of hospitalized patients with cirrhosis. We hypothesized that those with HRS would have the worst 90-day mortality.

## 2. Methods

### 2.1. Study Population and Setting

Between January 2013 and December 2014, consenting adult patients (age 18 years or older) who were hospitalized at Massachusetts General Hospital with cirrhosis and AKI (see “Definitions”) were enrolled in this prospective study. Massachusetts General Hospital is a 1008-bed academic tertiary care center with an active liver transplant program. Potential participants were excluded from this study if they previously received a renal transplant, if they were on renal replacement therapy at the time of admission, or if they were pregnant or nursing. Participants were followed during their inpatient admission and subsequently as outpatients after discharge.

### 2.2. Definitions

#### 2.2.1. Cirrhosis

The diagnosis of cirrhosis was based on clinical evaluation by a hepatologist using laboratory values, liver imaging, endoscopy, and (when available) liver biopsy.

#### 2.2.2. Acute Kidney Injury

The AKIN criteria were used to diagnose AKI, which required an absolute increase in serum creatinine of 0.3 mg/dL (26.4 *μ*mol/L) above baseline or an increase of serum creatinine to 150% of baseline within 48 hours [21]. The AKIN criterion for decline in urine output was not used in the initial diagnosis of AKI as it was felt to be unreliable in patients with ascites and without a bladder catheter.

#### 2.2.3. Classification of AKI

Participants were classified as having one of four types of AKI: (1) prerenal azotemia (PRA), (2) hepatorenal syndrome (HRS), (3) acute tubular necrosis (ATN), and (4) other causes. Participants were classified as having HRS based on the 2007 Ascites Club Criteria [14]. These include the following: (1) presence of cirrhosis and ascites, (2) serum creatinine greater than 1.5 mg/dL, (3) failure of improvement in serum creatinine below 1.5 mg/dL after administration of albumin and withdrawal of diuretics for 48 hours, (4) absence of nephrotoxic drugs, (5) absence of shock, and (6) absence of parenchymal renal disease. Parenchymal renal disease was defined as presence of abnormal kidneys on ultrasound, >500 mg proteinuria per day, presence of >50 RBCs per high-powered field of urinary sediment, or presence of granular casts on a nephrologist's review of the urinary sediment. Participants were classified as having PRA if they presented with AKI, a clinical history consistent with a prerenal state (such as bleeding or GI fluid losses), and their serum creatinine improved following the administration of volume and withdrawal of diuretics. Participants were diagnosed with ATN if they failed to meet criteria for PRA and HRS and had a clinical history consistent with tubular/parenchymal kidney injury. Participants were diagnosed with “other” causes of AKI if they had evidence of another process on serology or renal biopsy, such as glomerulonephritis.

Diagnoses of AKI were reviewed and confirmed independently by two study investigators. If a discrepancy in diagnosis was found, a third investigator reviewed the medical record and provided a tie-breaking diagnosis.

### 2.3. Data Collection and Management

All data was registered as part of the routine clinical care and collected from review of the electronic medical record. This included demographics, laboratory findings, radiology, procedural findings (including microscopic examination of urine sediment by a nephrologist), medical history, and medications. MELD and CLIF-SOFA scores were taken from the time of admission. The reference laboratory at the study site reported the lowest cutoff of urine sodium as “less than 10 mmol/L,” so this variable was dichotomized for analysis at this value. All values reported are taken from the time of enrollment unless otherwise noted. Study data were collected and managed using REDCap electronic data capture tools hosted at the Harvard Clinical and Translational Science Center [24]. Each participant also provided serum, plasma, and urine samples on enrollment and on days 5 and 30 after enrollment (as available) to create a biorepository for future evaluation of markers of kidney and liver injury.

Participants were treated as per local standard of care by the managing internists, hepatologists, and nephrologists on service. Members of the study team did not intervene in patient care. Management of AKI was done using guidelines and evidenced-based medicine at the discretion of treating clinicians, including (1) withdrawal of potential offending agents, such as diuretics, (2) empiric and culture based treatment of infections, (3) administration of volume, (4) reversal of underlying insults, such as endoscopic treatment of bleeding, (5) treatment of suspected HRS using albumin and midodrine/octreotide or vasopressors, and (6) use of intermittent hemodialysis or continuous venovenous hemofiltration for AKI refractory to medical management. Participants diagnosed with spontaneous bacterial peritonitis were given 1.5 g/kg albumin on day of diagnosis and 1 g/kg albumin 48 hours later along with antibiotics [25].

### 2.4. Statistical Analysis

Demographic and clinical characteristics of participants are presented as medians (quartile 1, quartile 3) or number (percentage) and compared between the four subgroups of AKI by one-way analysis of variance tests or Fisher's exact tests. These variables were also compared between those alive and those who died at 90 days using Mann-Whitney *U* tests for continuous variables and Fisher's exact test for categorical variables. Additional outcomes (need for dialysis, recovery from dialysis, creatinine at 90 days, and liver transplantation after enrollment) were analyzed by type of AKI in a similar fashion. Pairwise testing was also performed to compare outcomes between participants with HRS versus ATN.

Differences in the primary outcome of death by 90 days by type of AKI were visualized using a Kaplan-Meier curve and compared using a log-rank test. Cox proportional hazard models were used to create a multivariable model to predict death by 90 days by type of AKI. The subgroup of “other causes” of AKI was not analyzed in the multivariable model to better highlight the three subgroups affected by altered hemodynamics (PRA, HRS, and ATN). Because of sample size limitations, two prespecified models were selected for analysis using type of AKI, age, and either MELD or CLIF-SOFA score as predictors. The assumption of proportional hazards was tested for all models. Results of Cox proportional hazard models are summarized with hazard ratios and Wald asymptotic 95% confidence intervals. Four sensitivity analyses were performed: (1) excluding four participants who had previously received a liver transplant, (2) using a composite endpoint of death or liver transplant by 90 days, (3) reclassifying 10 participants with granular casts on sediment as having HRS if all other criteria were met, and (4) including infection as a subgroup of AKI (AKI with infection compared to three subgroups [PRA, HRS, and HRS] without infection). SAS version 9.4 (Gary, NC) was used for all analyses. Two-tailed *p* values < 0.05 were considered to indicate statistical significance.

### 2.5. Ethics Statement

This study was approved by the site's institutional review board and abides by the guidelines set forth by the Declaration of Helsinki. No donor organs were obtained from executed prisoners or other institutionalized persons at this liver transplant center. All participants (or their health care designee) provided written informed consent.

## 3. Results

### 3.1. General Demographics

**Figure 1:**
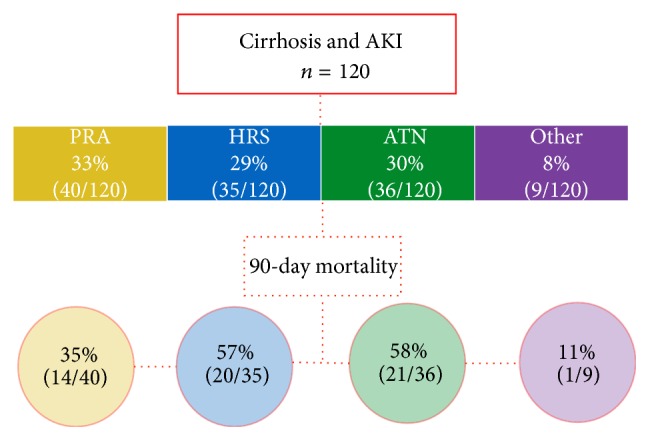
Distribution of participants and 90-day mortality. AKI (acute kidney injury), PRA (prerenal azotemia), HRS (hepatorenal syndrome), and ATN (acute tubular necrosis).

One hundred twenty participants with cirrhosis and AKI were analyzed in this study. Forty (33%) had PRA, 35 (29%) had HRS, 36 (30%) had ATN, and 9 (8%) had other causes of AKI (Figure 1). Of the other causes of AKI, 5 (56%) had evidence of glomerulonephritis. Ten participants who met all inclusion criteria for HRS (22%) were reclassified to acute tubular necrosis because of the presence of granular casts on urinary sediment.

Median (quartile 1, quartile 3) age of the entire cohort was 58 (50, 65) years. The majority of participants were male (71%), white race (93%), and of non-Hispanic ethnicity (87%). Median length of hospital admission was 16 (9, 24) days. Median time from admission to enrollment was 4 (3, 10) days. Median MELD score was 24 (18, 30) and median CLIF-SOFA score was 9 (6, 10). The most common etiologies of cirrhosis were alcoholic (30%), multifactorial (27%), and hepatitis C (20%). Eleven participants (9%) had stage I AKI, 23 participants (19%) had stage II AKI, and 86 participants (72%) had stage III AKI. Ninety-four participants (78%) received nephrology consultation. Thirty-eight participants (32%) required renal replacement therapy. Twenty-one participants (18%) went on to receive liver transplantation. Forty-nine participants (41%) received vasopressors while being hospitalized in the intensive care unit. Thirty-one participants with HRS (89%) were treated with midodrine and octreotide. Characteristics of all participants by type of AKI are presented in Table 1.

Across the entire cohort, 56 (47%) participants died by day 90 after enrollment. Participants who died had a significantly higher MELD score, CLIF-SOFA score, serum creatinine on enrollment, peak creatinine during their admission, INR, and total bilirubin and had a lower urine output and mean arterial pressure. Participants who died were more likely to receive nephrology consultation, receive dialysis, receive intravenous albumin, or be treated with midodrine, octreotide, or intravenous vasopressors (Table 2).

Among those with HRS, 13/15 (87%) who survived and 18/20 (90%) who died were treated with midodrine and octreotide (*p* = 1.00). Intravenous vasopressors were given to 6/15 (40%) who survived and 10/20 (50%) who died (*p* = 0.73).

### 3.2. Outcomes by Type of AKI

The primary and secondary outcomes were analyzed across all four types of AKI (PRA, HRS, ATN, and other) and in pairwise analysis between those with HRS and ATN (Table 3). There was a significant difference in death by 90 days across all four subgroups (*p* = 0.02) but not between HRS and ATN (20/35 [57%] versus 21/36 [58%]; *p* = 1.00). There was no significant difference in the need for dialysis (*p* = 0.13), recovery from dialysis (among survivors to 90 days; *p* = 0.16), and new liver transplant (*p* = 0.44). Among those who were alive and did not require dialysis, participants with HRS had a higher serum creatinine at 90 days than those with ATN (1.5 [1.2, 2.0] mg/dL versus 1.0 [0.8, 1.5] mg/dL; *p* = 0.01).

Overall, MELD score was significantly different across all four groups for all participants (*p* = 0.04), but not between HRS and ATN (24 [21,31] versus 26 [20,34]; *p* = 0.67). Among those who died, there was no significant difference in MELD score across all four groups (*p* = 0.90). CLIF-SOFA score was significantly different across all four groups for all participants (*p* = 0.01), but not between HRS and ATN (9 [8,10] versus 10 [7,12]; *p* = 0.17). Similarly, there was no significant difference in CLIF-SOFA scores among participants who died across all four subgroups (*p* = 0.84). Those with HRS were more likely to have a urine sodium less than 10 mmol/L compared to those with ATN (21/28 [75%] versus 5/19 [26%]; *p* = 0.01).

**Figure 2:**
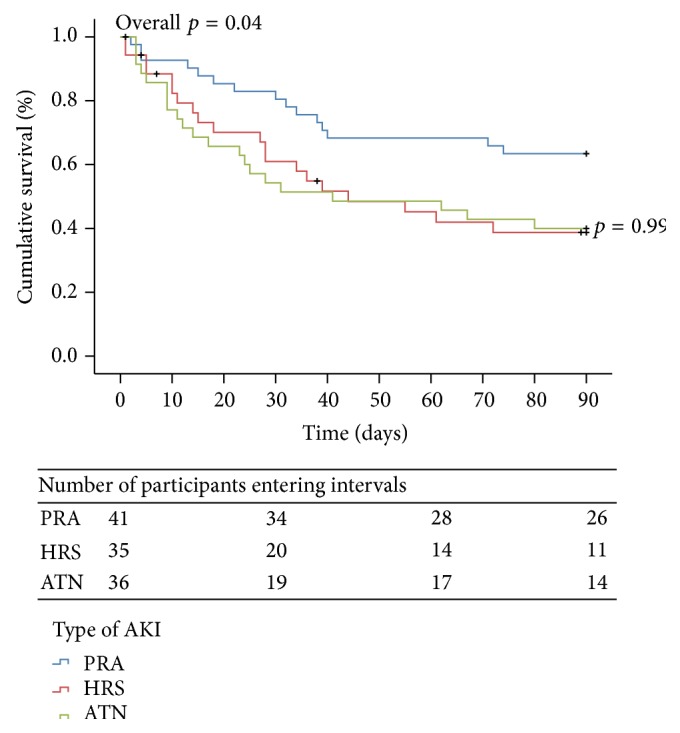
Ninety-day probability of survival of participants with cirrhosis by type of acute kidney injury. PRA (prerenal azotemia), HRS (hepatorenal syndrome), and ATN (acute tubular necrosis).

A Kaplan-Meier curve depicts survival through 90 days by type of AKI (PRA, HRS, and ATN) in Figure 2. Overall, there was a significant difference in survival between the three subgroups (*p* = 0.04). However, there was no difference in survival between HRS and ATN (*p* = 0.99). Mortality was significantly lower in PRA compared to HRS and ATN (*p* = 0.05 and *p* = 0.04, resp.).

In a sensitivity analysis using a combined composite endpoint of death or liver transplant by 90 days, results were similar to the primary outcome, with a significant difference across all three subgroups (*p* < 0.001), but not between HRS and ATN (*p* = 0.41). When examining those with AKI and infection, there was no difference in 90-day mortality between groups with infection and those without infection (*p* = 0.09 between all groups; *p* = 0.06 for AKI with infection versus PRA without infection; *p* = 0.39 for AKI with infection versus HRS without infection; *p* = 0.97 for AKI with infection versus ATN without infection).

Because 10 participants met HRS criteria but were classified as having ATN due to granular casts on urinary sediment, a sensitivity analysis was performed including them in the HRS subgroup. This resulted in 45 participants being classified as having HRS and reduced the number of those with ATN to 26. There was no change in the 90-day survival in this sensitivity analysis (*p* = 0.03 overall and *p* = 0.63 for HRS versus ATN).

In multivariable Cox proportional hazard models containing terms for type of AKI, age, and a prognostic score (either MELD score [Model 1] or CLIF-SOFA score [Model 2]), there was a trend towards lower risk of death with a diagnosis of PRA compared to ATN (Model 1 HR: 0.52, 95% CI [0.25–1.07]; *p* = 0.08, and Model 2 HR: 0.55, 95% CI [0.27–1.14]; *p* = 0.11) while risk of death for HRS did not differ from ATN (Model 1 HR: 0.97, 95% CI [0.52–1.79]; *p* = 0.91, and Model 2 HR: 1.03, 95% CI [0.55–1.93]; *p* = 0.93). Age was associated with increased risk of death (Model 1 HR: 1.05, 95% CI [1.02–1.09]; *p* = 0.01, and Model 2 HR: 1.05, 95% CI [1.02–1.09]; *p* = 0.001). Higher MELD or CLIF-SOFA scores were associated with increased risk of death (Model 1 HR: 1.04, 95% CI [1.01–1.08]; *p* = 0.04, and Model 2 HR: 1.15, 95% CI [1.02–1.30]; *p* = 0.02).

### 3.3. Hepatorenal Syndrome Diagnostic Criteria

**Figure 3:**
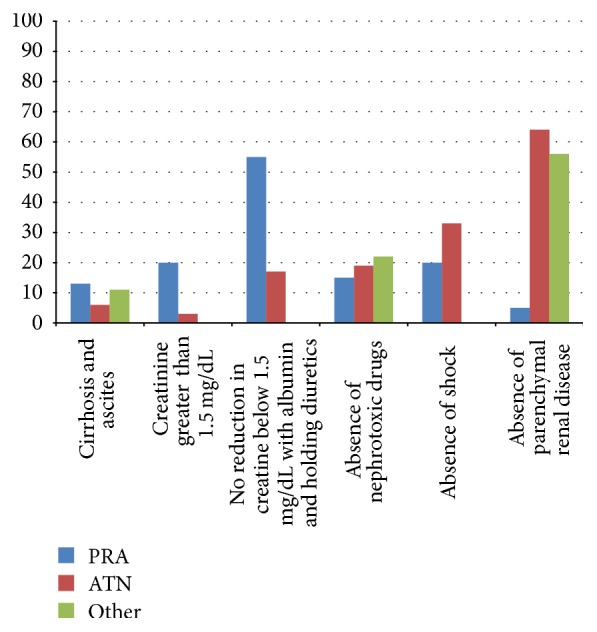
Percentage of participants with acute kidney injury who failed to satisfy Ascites Club Criteria for hepatorenal syndrome. PRA (prerenal azotemia), ATN (acute tubular necrosis).

Each of the six components of the Ascites Club Criteria was analyzed in the PRA, ATN, and “other” AKI subgroups to determine how each participant failed to meet diagnostic criteria for HRS (Figure 3). In the ATN subgroup, participants most commonly had evidence of parenchymal renal disease (55%) or shock (33%). In the PRA subgroup, participants most commonly had a serum creatinine lower than 1.5 mg/dL (20%), had a reduction of creatinine below 1.5 mg/dL with albumin and holding diuretics (55%), or had shock (20%). In the subgroup of “other” causes of AKI, participants most commonly had evidence of parenchymal renal disease (56%) or exposure to nephrotoxic drugs (22%).

## 4. Discussion

Our results suggest that, among those with cirrhosis and AKI, 90-day mortality is the same between those with HRS and ATN in crude analysis and after adjusting for age and a cirrhosis-specific prognostic assessment (MELD or CLIF-SOFA score). Those with PRA have a better 90-day mortality rate compared to HRS and ATN. In univariate analysis, several factors related to liver and kidney function were associated with mortality, including serum creatinine, need for dialysis, urine output, international normalized ratio, and total bilirubin. Many of these factors are reflected in widely used risk scores [8, 9, 26, 27], thus highlighting their importance in prognosis and transplant allocation.

Interestingly, our results differ from the work done by Martin-Llahi and colleagues in a similar study [13]. In their single-center, prospective cohort study of inpatients with cirrhosis and AKI, these investigators found that 90-day mortality was highest in those with HRS. Those with parenchymal nephropathy had the lowest mortality, and those with hypovolemia-mediated AKI (i.e., PRA) fell in between these two groups. We do not believe that it is biologically plausible for those with PRA to have worse mortality than those with ATN. However, there may be several reasons for the differences between our studies. First, Martin-Llahi and colleagues originally classified AKI into four subgroups, including an additional category of those with AKI mediated by infection. Because infection can be a trigger of PRA (through volume depletion from fluid losses and decreased effective circulating volume), HRS (as can be the case with spontaneous bacterial peritonitis) [25, 28], and ATN (through systemic inflammation or shock) [29], we elected to include those with infection within a PRA/HRS/ATN scheme. An analysis of 90-day mortality that stratified AKI by presence of infection did not show a significant difference between groups in our cohort. Nevertheless, it may be useful to consider infection as an independent risk factor for mortality with AKI in cirrhosis [13, 30]. Second, there are likely variations in populations between our two studies, potentially due to differences in patient demographics and local practice patterns (e.g., terlipressin, an approved treatment for HRS, is not available in the United States but is commonly employed in Europe) or in the definition of HRS and AKI (the most recent Ascites Club Criteria and AKIN definition of AKI were not available at the time of Martin-Llahi et al.'s study recruitment). These differences are highlighted in HRS incidences (29% in our study versus 51% in their cohort) as well as overall 90-day mortality (47% versus 70%, resp.). These differences, along with variable incidence and survival rates in the literature, suggest a critical need for a multicenter study to better describe the importance of type of AKI in cirrhosis.

One important area of AKI in cirrhosis is the role of the nephrologist, though to our knowledge no prior studies have examined this aspect of care. In our sample, 78% of participants received a nephrology consultation. The nephrologist can offer careful examination of the urine sediment. Coarse pigmented granular casts are a hallmark of ATN; their absence has an excellent negative predictive value in distinguishing prerenal disease (such as PRA and HRS) from intrinsic renal insults [31]. Although one criterion for HRS is the “absence of parenchymal renal injury,” only significant proteinuria, hematuria, and ultrasonographic abnormalities are listed as examples [14]. The presence of granular casts is a by-product of the pathophysiology of parenchymal injury, yet it has not been listed in the diagnostic algorithm for HRS for two iterations (it was listed as “minor criteria” in the 1979 Sassari criteria) [15]. We believe that the presence of granular casts on a nephrologist's review of sediment should be considered in the diagnosis of HRS as a tool to guide therapeutic approach to this population.

While we consider HRS a diagnosis of exclusion, it remains possible that overlap between the clinical syndromes of HRS and ATN partially explains their similar mortality rates in this study. Increasingly, clinicians are recognizing the presence of bilirubin nephropathy (sometimes referred to as cholemic nephrosis) as a consequence of advanced cirrhosis. The pathophysiology of bilirubin nephropathy is similar to that of myoglobinuria or rhabdomyolysis, where high serum bilirubin levels are directly toxic to renal tubules when filtered, resulting in a clinical picture consistent with ATN [32]. In an autopsy series in patients with jaundice, van Slambrouck et al. noted that 11/13 of those with a clinical diagnosis of HRS had evidence of tubular bile casts on histologic examination of the kidney [33]. Our current understanding of HRS suggests that it is a hemodynamically driven process without intrinsic renal damage; thus it is possible that some patients with HRS have a secondary insult of ATN. The absence of a diagnostic test linked to the biology of disease and nonspecific clinical criteria do not allow the clinician to simultaneously diagnose a patient with ATN and HRS, even though it is possible that multiple insults may explain a patient's clinical status. Martin-Llahi et al. excluded 8% of their cohort from final analysis due to a mixed etiology of AKI, which further supports this idea [13].

Regardless of the nuances in diagnostic differences between HRS and ATN, our results confirm that those with cirrhosis and AKI represent a critically ill population. Instead of debating the current framework on which we diagnose HRS, it is more important for the clinician to determine whether there is a rapidly reversible, volume-responsive injury (such as PRA) or an insult like HRS or ATN that requires close supportive care. Small trials suggest that vasoconstrictors, albumin, and (in certain circumstances) transjugular intrahepatic shunt placement may be beneficial in HRS [34–41]. Increase in mean arterial pressure (via midodrine, octreotide, or vasopressors) has been linked to improved outcomes in HRS. Maintaining acceptable blood pressure and euvolemic volume status is within the guidelines of managing ATN as well [29]. Empiric use of available therapies, along with early and appropriate use of dialysis as a bridge to transplantation, remains the cornerstone of care for all those with AKI that is not responsive to volume repletion.

Our study should be interpreted within the context of its limitations. This was a single-center trial and may not be generalizable to the experience at other liver transplant centers. However, no multicenter studies have been published examining this subject; thus we believe that our results represent an important contribution to the literature. Similarly, our cohort was predominantly male, white race, and of non-Hispanic ethnicity, limiting generalizability. However, prior studies on cirrhosis and AKI either are similarly homogenous or do not report these patient characteristics, and our results describe the first American cohort in this area. This study was unable to use urine output as part of the AKIN criteria due to unreliable recorded data, a limitation that is commonly reported across many different clinical and research settings. Given our sample size, we were limited in methods of constructing a multivariable model to explain 90-day mortality and were unable to perform a model building process that allowed for screening or selection of a list of candidate variables. However, we feel that our model reflects a pragmatic scientific approach, as age and prognostic assessments like MELD and CLIF-SOFA scores have been well established as important contributors to mortality.

## 5. Conclusions

We present one of the only studies of US cohort examining implications of the etiology of AKI in cirrhosis. We showed a similar 90-day mortality rate between individuals with HRS and ATN, which was higher compared to the mortality in those with PRA. While there may be some differences in patient characteristics or in the diagnostic approach to HRS from center to center, it remains clear that AKI in cirrhosis portends a high mortality. Further study and multicenter clinical trials are needed to help clinicians better diagnose and improve outcomes in this critically ill group of patients.

## Figures and Tables

**Table 1:** Demographics and clinical characteristics.

	PRA	HRS	ATN	Other	*p* value
	(*n* = 40)	(*n* = 35)	(*n* = 36)	(*n* = 9)	
Age (years)	58 (49.5, 66)	57 (49, 65)	60 (51, 63)	57 (51, 62)	0.80
Male sex (%)	26 (65%)	25 (71%)	26 (72%)	8 (89%)	0.60
White race (%)	34 (85%)	34 (97%)	34 (94%)	9 (100%)	0.49
Non-Hispanic ethnicity (%)	31 (78%)	30 (86%)	34 (94%)	9 (100%)	0.23
Body mass index (kg/m^2^)	28.8 (26.5, 34.6)	28.3 (23.7, 32.0)	27.4 (22.5, 32.8)	26.9 (25.1, 31.0)	0.38
Presence of infection (%)	15 (38%)	11 (31%)	18 (50%)	1 (11%)	0.14
Comorbidities (%)					
Diabetes mellitus	11 (29%)	10 (29%)	11 (31%)	4 (8%)	0.81
Chronic kidney disease	7 (18%)	14 (41%)	10 (28%)	7 (78%)	0.01
Cardiovascular disease	8 (20%)	6 (17%)	7 (19%)	3 (33%)	0.73
Hypertension	17 (43%)	14 (40%)	14 (40%)	3 (33%)	0.99
Etiology of cirrhosis (%)					0.24
Hepatitis C	11 (28%)	4 (11%)	7 (19%)	2 (22%)	
Alcohol	12 (30%)	12 (35%)	12 (33%)	0 (0%)	
Nonalcoholic steatohepatitis	3 (8%)	4 (11%)	5 (14%)	1 (11%)	
Multifactorial	12 (30%)	11 (31%)	6 (17%)	3 (33%)	
Other	2 (5%)	4 (11%)	6 (17%)	3 (33%)	
Prior complications of cirrhosis (%)					
Ascites requiring prior paracentesis	14 (36%)	27 (82%)	12 (33%)	4 (44%)	<0.001
Encephalopathy	14 (35%)	20 (57%)	12 (33%)	3 (33%)	0.15
Gastrointestinal bleeding	7 (18%)	8 (23%)	6 (17%)	0 (0%)	0.52
Spontaneous bacterial peritonitis	4 (10%)	7 (20%)	3 (8%)	0 (0%)	0.35
Portosystemic shunt	3 (8%)	2 (6%)	4 (11%)	2 (22%)	0.42
Prior liver transplantation (%)	2 (5%)	1 (3%)	0 (0%)	1 (11%)	0.57
MELD score (admission)	19 (17, 28)	24 (21, 31)	26 (20, 34)	20 (18, 21)	0.04
CLIF-SOFA score (admission)	8 (6, 9)	9 (8, 10)	10 (7, 12)	6 (5, 7)	0.01
Nephrologist consulted (%)	23 (58%)	31 (89%)	33 (92%)	6 (67%)	<0.001
AKI stage I or II (%)/stage III (%)	15 (38%)/25 (63%)	9 (26%)/26 (75%)	7 (19%)/29 (81%)	3 (33%)/6 (67%)	0.34
Medications received (%)					
Intravenous albumin	28 (70%)	34 (97%)^*∗*^	29 (81%)	4 (44%)	<0.001
Midodrine	15 (38%)	31 (89%)	25 (69%)	2 (22%)	<0.001
Octreotide	21 (53%)	31 (89%)	24 (67%)	1 (11%)	<0.001
Intravenous vasopressor	13 (33%)	15 (43%)	19 (53%)	2 (22%)	0.21
Laboratory values/vital signs					
Mean arterial pressure (mmHg)	78 (73, 85)	74 (69, 80)	73 (68, 80)	86 (79, 88)	0.07
Urine output (mL/24 hours)^*∗∗*^	763 (475, 1125)	488 (325, 750)	625 (300, 1095)	873 (525, 1450)	0.23
Enrollment creatinine (mg/dL)	1.4 (1.2, 1.8)	2.7 (2.0, 3.0)	2.3 (1.9, 3.6)	2.3 (2.0, 3.7)	<0.001
Peak creatinine (mg/dL)	1.9 (1.6, 2.6)	3.4 (2.7, 4.7)	3.5 (2.5, 6.8)	4.1 (2.3, 4.5)	<0.001
Sodium (mEq/L)	133 (130, 137)	131 (128, 136)	136 (132, 141)	136 (129, 140)	0.10
White blood count (K/uL)	7.2 (4.3, 10.6)	7.0 (4.8, 10.6)	9.1 (5.5, 15.7)	6.4 (4.1, 8.8)	0.10
Hemoglobin (g/dL)	8.3 (8.0, 9.5)	9.0 (7.9, 10.0)	8.6 (7.9, 9.4)	8.9 (8.4, 11.1)	0.44
Platelets (K/uL)	89 (57, 124)	77 (58, 101)	64 (47, 125)	93 (77, 143)	0.50
Albumin (g/dL)	2.9 (2.5, 3.6)	3.5 (3.2, 3.7)	2.8 (2.7, 3.2)	2.8 (2.5, 4.1)	0.01
International normalized ratio (INR)	1.7 (1.3, 2.0)	1.8 (1.5, 2.1)	1.9 (1.5, 2.3)	1.4 (1.2, 1.5)	0.01
Total bilirubin (mg/dL)	3.8 (1.7, 8.2)	5.0 (2.2, 11.0)	9.6 (2.5, 22.2)	2.0 (1.1, 3.8)	0.06
Urine sodium <10 mmol/L (%)^*∗∗∗*^	9 (43%)	21 (75%)	5 (26%)	2 (33%)	<0.001

All values were taken at time of study enrollment unless otherwise noted. Continuous variables presented as median (quartile 1, quartile 3).

PRA: prerenal azotemia, HRS: hepatorenal syndrome, and ATN: acute tubular necrosis.

^*∗*^One participant received red blood cell transfusion instead of albumin.

^*∗∗*^ *n* = 36 for PRA, *n* = 34 for HRS, *n* = 30 for ATN, and *n* = 8 for other.

^*∗∗∗*^ *n* = 21 for PRA, *n* = 28 for HRS, *n* = 19 for ATN, and *n* = 6 for other.

**Table 2:** Relationship of variables to death at 90 days for all participants^*∗*^.

	Alive	Died	*p* value
	(*n* = 64)	(*n* = 56)	
Age (years)	58 (48, 64)	59 (53, 66)	0.12
Male sex (%)	43 (69%)	41 (73%)	0.69
White race (%)	58 (91%)	53 (95%)	0.44
Non-Hispanic ethnicity (%)	53 (82%)	51 (91%)	0.34
Body mass index (kg/m^2^)	27.2 (23.6, 33.2)	29.0 (25.5, 32.2)	0.47
Presence of infection (%)	20 (31%)	25 (45%)	0.14
Other medical problems (%)			
Diabetes mellitus	19 (31%)	17 (31%)	1.00
Chronic kidney disease	23 (37%)	15 (27%)	0.33
Cardiovascular disease	14 (22%)	10 (18%)	0.65
Hypertension	27 (43%)	21 (38%)	0.58
Etiology of cirrhosis^*∗∗*^	—	—	0.46
Prior complications of cirrhosis (%)			
Any ascites	41 (64%)	33 (59%)	0.58
Ascites requiring paracentesis	28 (45%)	29 (53%)	0.46
Encephalopathy	27 (42%)	22 (39%)	0.85
Gastrointestinal bleeding	8 (13%)	13 (23%)	0.15
Spontaneous bacterial peritonitis	8 (13%)	6 (11%)	0.78
Portosystemic shunt	7 (11%)	4 (8%)	0.54
MELD score (admission)	23 (18, 29)	29 (23, 36)	<0.001
CLIF-SOFA score (admission)	8 (5, 10)	9 (8, 11)	0.01
Nephrologist consulted (%)	42 (66%)	51 (91%)	<0.001
AKI stage I or II (%)/stage III (%)	23 (36%)/41 (64%)	11 (20%)/45 (80%)	0.07
Required dialysis (%)	14 (22%)	24 (43%)	0.02
Medications received (%)			
Intravenous albumin	45 (70%)	50 (89%)	0.01
Midodrine	28 (44%)	45 (80%)	<0.001
Octreotide	32 (50%)	45 (80%)	<0.001
Intravenous vasopressor	20 (31%)	30 (54%)	0.02
Laboratory values/vital signs			
Mean arterial pressure (mmHg)	78 (71, 84)	74 (68, 80)	0.03
Urine output (mL/24 hours)^*∗∗∗*^	850 (500, 1200)	450 (275, 750)	<0.001
Enrollment creatinine (mg/dL)	1.8 (1.3, 2.4)	2.6 (1.9, 3.5)	<0.001
Peak creatinine (mg/dL)	2.3 (1.8, 3.9)	3.4 (2.8, 4.7)	<0.001
Sodium (mEq/L)	133 (129, 137)	134 (130, 139)	0.41
White blood count (K/uL)	10.1 (6.9, 16.9)	8.2 (5.3, 13.5)	0.09
Hemoglobin (g/dL)	8.3 (7.8, 9.5)	8.9 (8.1, 10.1)	0.09
Platelets (K/uL)	88 (59, 124)	72 (48, 104)	0.20
Albumin (g/dL)	2.9 (2.5, 3.5)	3.3 (2.8, 3.6)	0.06
International normalized ratio (INR)	1.6 (1.3, 2.0)	1.9 (1.5, 2.1)	0.02
Total bilirubin (mg/dL)	3.7 (1.5, 8.7)	7.1 (3.0, 19.9)	0.001
Urine sodium < 10 mmol/L (%)^*∗∗∗∗*^	15 (39%)	22 (61%)	0.10

All values were taken at time of study enrollment unless otherwise noted. Continuous variables presented as median (quartile 1, quartile 3).

AKI: acute kidney injury.

^*∗*^Three participants who were lost to follow-up were included in the alive category.

^*∗∗*^Subcategories the same as Table 1 (hepatitis C, alcohol, nonalcoholic steatohepatitis, multifactorial, other).

^*∗∗∗*^ *n* = 108 total.

^*∗∗∗∗*^ *n* = 74 total.

**Table 3:** Outcomes and variables by type of acute kidney injury.

	PRA	HRS	ATN	Other	*p* value	*p* value
	*n* = 40	*n* = 35	*n* = 36	*n* = 9	(overall)	(HRS versus ATN)
Death by 90 days (%)	14 (35%)	20 (57%)	21 (58%)	1 (11%)	0.02	1.00
Required dialysis (%)	8 (20%)	12 (34%)	16 (44%)	2 (22%)	0.13	0.47
Recovered from dialysis (%)^*∗*^	4 (44%)	1 (8%)	3 (19%)	1 (50%)	0.16	0.61
Creatinine at 90 days (mg/dL)^*∗∗*^	1.0 (0.8, 1.3)	1.5 (1.2, 2.0)	1.0 (0.8, 1.5)	1.6 (1.2, 2.1)	0.01	0.02
Received liver transplant (%)	6 (15%)	9 (26%)	5 (15%)	1 (11%)	0.44	0.24

Continuous variables presented as median (quartile 1, quartile 3).

PRA: prerenal azotemia, HRS: hepatorenal syndrome, ATN: acute tubular necrosis, MELD: Model for End-Stage Liver Disease, and CLIF-SOFA: Chronic Liver Failure-Sequential Organ Failure Assessment.

^*∗*^Among those who required dialysis (*n* = 9 for PRA, *n* = 12 for HRS, *n* = 16 for ATN, and *n* = 2 for other).

^*∗∗*^Among those who were alive and were not requiring dialysis at 90 days (*n* = 24 for PRA, *n* = 13 for HRS, *n* = 10 for ATN, and *n* = 6 for other).

## List of Abbreviations

AKI: Acute kidney injury
PRA: Prerenal azotemia
ATN: Acute tubular necrosis
HRS: Hepatorenal syndrome
MELD: Model for End-Stage Liver Disease
CLIF-SOFA: Chronic Liver Failure-Sequential Organ Failure Assessment
AKIN: Acute Kidney Injury Network.
